# Emodin Induced Necroptosis and Inhibited Glycolysis in the Renal Cancer Cells by Enhancing ROS

**DOI:** 10.1155/2021/8840590

**Published:** 2021-01-19

**Authors:** Ke-jie Wang, Xiang-yu Meng, Jun-feng Chen, Kai-yun Wang, Cheng Zhou, Rui Yu, Qi Ma

**Affiliations:** ^1^Translational Research Laboratory for Urology, The Key Laboratory of Ningbo City, Ningbo First Hospital, Ningbo, Zhejiang 315010, China; ^2^Ningbo Clinical Research Center for Urological Disease, Ningbo, Zhejiang 315010, China; ^3^Comprehensive Urogenital Cancer Center, Ningbo First Hospital, Ningbo, Zhejiang 315010, China; ^4^Department of Urology, Ningbo First Hospital, Ningbo, Zhejiang 315010, China; ^5^Medical School, Ningbo University, #818 Fenghua Road, Ningbo, Zhejiang 315211, China

## Abstract

Renal cell carcinoma (RCC) is a tumor with unpredictable presentation and poor clinical outcome. RCC is always resistant to chemotherapy and radiation, and weakly sensitive to immunotherapeutic agents. Therefore, novel agents and approaches are urgently needed for the treatment of RCC. Emodin, an anthraquinone compound extracted from rhubarb and other traditional Chinese herbs, has been implicated in a wide variety of pharmacological effects, such as anti-inflammatory, antiviral, and antitumor activities. However, its role in RCC remains unknown. In this study, we found that emodin effectively killed renal cancer cells without significant toxicity to noncancerous cell HK-2. Flow cytometry assay with Annexin V-FITC and PI demonstrated that emodin induces necroptosis, but not apoptosis, in renal cancer cells. Meanwhile, the phosphorylation levels of RIP1 and MLKL, the key necroptosis-related proteins, were significantly increased. To explore how emodin inhibits kidney tumor growth, we tested reactive oxygen species (ROS) levels and found that the levels of ROS increased upon emodin treatment in a dose-dependent manner. Further studies demonstrated that emodin induces necroptosis through ROS-mediated activation of JNK signaling pathway and also inhibits glycolysis by downregulation of GLUT1 through ROS-mediated inactivation of the PI3K/AKT signaling pathway. Our findings revealed the potential mechanisms by which emodin suppresses renal cancer cell growth and will help develop novel therapeutic approaches for patients with JNK- or PI3K/AKT-dysregulated renal cancer.

## 1. Introduction

Renal cell carcinoma (RCC) is a common urologic tumor derived from the highly heterogeneous epithelium of renal tubules [1]. For patients who received curative surgical resection, the tumor recurrence is 40% and for metastatic patients, the five-year survival rate is only 12% [2]. Moreover, RCC is also resistant chemotherapy and radiation and responds poorly to immunotherapeutic agents, such as interleukin- (IL-) 12 and interferon- (INF-) *α* [3]. To date, RCC is still a tumor with unpredictable presentation and poor clinical outcome. Therefore, novel drugs are urgently desired for the treatment of RCC.

Reactive oxygen species (ROS) refers to a series of intermediate products in the oxidation-reduction process, including superoxide radicals, hydrogen peroxide, and hydroxyl radicals. Intracellular ROS plays important roles in a variety of normal biochemical functions and abnormal pathological processes [4, 5]. Recently, ROS has been reported to improve the antitumor efficacy of chemotherapeutic drugs through enhancing their cytotoxic effects. Previous studies showed that intercellular ROS generation induced by antitumor drugs could trigger apoptosis in cancer cells [6–8]. Zou et al. verified that Auranofin can induce apoptosis of gastric cancer cells by increasing endoplasmic reticulum stress which depends on the increasing intracellular ROS levels [9]. Another study in colorectal cancer cells also found that induction of ROS overload by Alantolactone can promote oxidative DNA damage and apoptosis [10]. Although the relationship between apoptosis and ROS has been well studied [11], other antitumor mechanisms of ROS remain to be explored.

Emodin (1,3,8-trihydroxy-6-methylindole), a natural terpenoid, is mainly found in traditional Chinese herbal medicines and derived from rhizomes and roots [12]. The molecular structure of emodin is similar to that of 2,3-dimethoxy-1,4-naphthoquinone (DMNQ). Because of its properties of hydroquinone and derived hydroquinone, similar to mitochondrial ubiquinone, it can transfer electrons to produce ROS [13]. A growing number of studies have reported that emodin shows striking antitumor effects [14]. For example, two studies revealed that emodin could increase the cisplatin-induced cytotoxicity against ovarian cancer and bladder cancer cells through ROS-related downregulation of MRP1 [15, 16]. Besides, in lung cancer, emodin increases expression of insulin-like growth factor-binding protein 1 through the activation of MEK/ERK/AMPK*α* and interaction of PPAR*γ* and Sp1 [17]. However, the antitumor effects of emodin in renal cancer is still uncovered. Necrosis has for a long time been considered exclusively as an accidental mode of cell death without any underlying coordinated program of signaling events. More recently, breakthrough discoveries have shown that regulated forms of necrosis also exist [18]. For example, necroptosis is one of the most well-studied forms of necrosis: its activation through the formation of RIP3 and RIP1 complexes, recruiting downstream MLKL to phosphorylate and localize to the cell membrane, causing cell membrane rupture, ultimately leading to cell death [19]. In our study, we found that emodin can kill renal cancer cells by inducing necroptosis, but not apoptosis, and inhibiting aerobic glycolysis. It provides a new insight into the mechanism underlying emodin killing cancer cells and helps develop novel therapeutic approaches for patients with RCC.

## 2. Materials and Methods

### 2.1. Reagents

Emodin was purchased from Beijing Century Aoke Biology Research Company. N-acetyl-cysteine, glucose-6-phosphate, ATP and LDH measurement kits were from the Beyotime Biotech company. The pyruvate measurement kit was from the Solarbio company. JNK inhibitor SP600125 and AKT activator SC79 were from MedChemExpress. Anti-RIP3 (ab56164), anti-phospho-RIP3 at Ser227 (ab209384), anti-MLKL (ab184718), caspase 8 (ab25901), caspase 3 (ab32351) and anti-phospho-MLKL at Ser358 (ab187091), and anti-GAPDH (ab181602) antibodies were purchased from the Abcam company (Cambridge, MA). Caspase 9 (#9502), anti-RIP1 (#4926), anti-phospho-RIP1 at Ser166 (#65746), anti-JNK (#9252), and anti-phospho-JNK at Thr183/Tyr185 (#4668) antibodies were from the cell signaling company (Danvers, MA).

### 2.2. Cell Lines and Culture

HK-2, ACHN, CaKi, 786-O, and OS-RC-2 cells were obtained from Shanghai Institute of Cell Biology, Chinese Academy of Sciences (Shanghai, China). These cells were cultured in suitable medium containing 10% fetal bovine serum. The cells were maintained at 37°C and 5% CO_2_ in a humid environment, and the cells in the mid-log phase were used in the experiments.

### 2.3. Cell Viability Assay

The cells were plated into 96-well plates (the cell number: 2x10^5^/well, the volume: 200 *μ*L/well) and incubated overnight. Then, different concentrations of emodin were added for 24 h. A total of 10 *μ*L of CCK-8 (Dojindo, Japan) solution was added to each well and incubated for 2 to 4 h. The absorbance was measured at 450 nm by using the iMark™ Microplate Reader (Bio-Rad, US).

### 2.4. Measuring Reactive Oxygen Species (ROS)

Mitochondrial ROS was measurement by the dye MitoSox (red Mitochondrial superoxide indicator, ThermoFisher, USA). Cells underwent with increasing doses of emodion for 24 h and further administered 5 *μ*M Mitosox for 30 min. The images of the red fluorescence of in the cells were acquired by Nikon Ti-U fluorescence microscope (Nikon Ti-U, Japan). Flow cytometry (Beckman Coulter, Fullerton, CA, USA) was used for the quantitative analysis of Mitosox.

The redox-sensitive dye DCFH-DA (Beyotime Biotech, Nanjing, China) was used to evaluate the levels of overall ROS. Briefly, the cells were incubated with increasing doses of emodion for 24 h and stained with 10 *μ*M DCFH-DA in the dark for 30 min. The images of the green fluorescence of the oxidized product dichlorofluorescin (DCF) in the cells were acquired by Nikon Ti-U fluorescence microscope. Flow cytometry (Beckman Coulter, Fullerton, CA, USA) was used for the quantitative analysis of DCF.

### 2.5. Necroptosis Assay by Lactate Dehydrogenase (LDH) Release

Cells were seeded onto a 96-well microplate and cultured for 24 hours. The lactate dehydrogenase cytotoxicity assay kit (Beyotime Biotech, Nanjing, China) was used to detect cell mortality after treatment with emodin. The detection method was performed according to the instructions of the kit. The absorbance value of each sample was read at 490 nm (iMark™ Microplate Reader, Bio-Rad, US).

### 2.6. Glucose-6-Phosphate (G-6-P) Examination

The glucose-6-phosphate examination was performed according to manufacturer instructions of the Beyotime Biotech company (Nanjing, China). Briefly, about 1 × 10^6^ cells per well were seeded in six-well plates, being treated with or without targeted compounds. Then, the cells were washed and harvested. The collected cells were lysed in G-6-P extracting solution, then 12,000 g, centrifuged at 4°C for 5-10 minutes, and the supernatant was taken as a sample to be tested and stored in an ice bath for later use. The samples were mixed with G-6-P reaction buffers and incubated at 37° C for 30 minutes in the dark. The readings were taken at 450 nm (iMark™ Microplate Reader, Bio-Rad, US). The results of different groups were all normalized with their own protein content.

### 2.7. Pyruvate (PA) Measurement

The PA examination was performed according to the manufacturer's instructions of the Solarbio company (Beijing, China). In brief, the cells were washed and harvested. The collected cells were lysed in assay buffer by repeated sonification (ice bath, power 20% or 200 W, ultrasound 3 s, interval 10 s, repeat 30 times). Then the sample was allowed to stand for 30 min, 8000 g, and centrifuged at room temperature for 10 min, and the supernatant was taken for testing. For the measurement of the concentration of pyruvate, the samples were mixed with PA reaction buffers and read at absorbance 520 nm in a microplate reader (iMark™ Microplate Reader, Bio-Rad, US). The results of different groups were all normalized with their own protein content.

### 2.8. ATP Measurement

ATP was measured using an ATP measurement kit (Beyotime Biotech, Nanjing, China) according to the manufacturer's instructions. Briefly, about 1 × 10^6^ cells per well were seeded in six-well plates, being treated with or without targeted compounds. Then, the cells were washed and harvested. The collected cells were lysed in ATP extracting solution, then 12,000 g, centrifuged at 4° C for 5 minutes, and the supernatant was taken as a sample to be tested, and stored in an ice bath for later use. The samples were mixed with ATP reaction buffers and the RLU values were determinated by a luminometer (SpectraMax iD3, Molecular Devices, USA). The results of different groups were all normalized with their own protein content.

### 2.9. Annexin V-Fluorescein/Propidium Iodide Double-Staining Assay

Loss of cell membrane integrity was assessed by the Annexin V-fluorescein (FITC)/propidium iodide (PI) double-staining assay (Lianke Biotechnology Corporate Limited, China). In brief, the cells were harvested and resuspended in 500 *μ*L 1× Binding Buffer. And the cell suspension was added with 10 *μ*L PI and 5 *μ*L Annexin V-FITC. After incubation for 15 min at room temperature in the dark, the cells were subjected to analysis on a FACScan flow cytometer (Becton Dickinson, USA). Data were analyzed with the FlowJo software (BD, USA).

### 2.10. Western Blot

#### 2.10.1. Total Protein Extraction

Cells were lysed in RIPA buffer supplemented with 1% protease inhibitors and 1% phosphatase inhibitor, according to the manufacturer's protocols. After centrifugation (14,000 rpm, 30 min), the supernatant fraction was collected and the protein concentration was quantitated by BCA Protein Assay Kit (Beyotime Biotech, Nanjing, China).

#### 2.10.2. Membrane Protein Extraction

Membrane protein extraction was performed using the cell membrane protein extraction kit (Beyotime Biotech, Nanjing, China), according to the manufacturer's protocols.

An equal amount of protein was separated on SDS-PAGE gel and electrotransferred to the PVDF membrane. After blocking with TBS containing 5% nonfat milk and 0.1% Tween-20 for 2 h, the membrane was incubated with the primary antibody at 4°C overnight; then the appropriate HRP-conjugated secondary antibody was applied and detected by chemiluminescence.

### 2.11. Statistical Analysis

All experiments were repeated three times. GraphPad Prism 5 (GraphPad Software, USA) was used for statistical analysis. The data in all figures (including supplementary figures) are shown as means ± SEM. ANOVA was used to evaluate the differences between groups. Two-sided Student's *t*-test was used to compare the differences between two groups. Differences were considered significant if *p* < 0.05, ∗*p* < 0.05, ∗∗*p* < 0.01, and ∗∗∗*p* < 0.001.

## 3. Results

### 3.1. Renal Carcinoma Cells Were Sensitive to Emodin

**Figure 1:**
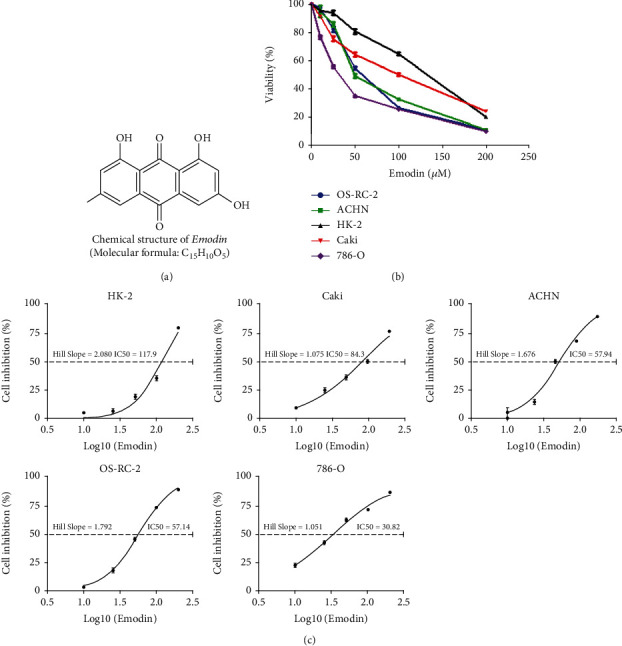
Emodin inhibited the viability of renal carcinoma cells. (a) The chemical structure of emodin. (b) Dose-dependent effects of emodin on renal cancer cells and renal tubular epithelial cell viability as determined by CCK-8 assays at 24 h. (c) The inhibitory effect of emodin on renal cancer cells and renal tubular epithelial cell proliferation as detected by CCK-8 assays after 24 h of treatment.

The chemical structure of emodin is indicated in Figure 1(a). To determine the effects of emodin on the viability of renal cancer cells, Cell Counting Kit-8 (CCK-8) assay was performed in 4 kidney cancer cells, including Caki, ACHN, 786-0, and OS-RC-2, as well as in noncancerous cells HK-2. The results showed that emodin significantly attenuated the survival rate of renal carcinoma cells in a dose-dependent manner (Figure 1(b)). Specifically, we found that the half maximal inhibitory concentration (IC50) of emodin at 24 h was 84.30, 57.94, 57.14, and 30.82 *μ*M in Caki, ACHN, 786-0, and OS-RC-2, respectively (Figure 1(c)). However, the HK-2 cells have much higher IC50 (117.9 *μ*M) values than kidney cancer cells (Figure 1(c)). Therefore, we selected OS-RC-2 and 786-0 cell lines for subsequent analyses as they are the most sensitive cell lines to emodin treatment.

### 3.2. Necroptosis Was the Main Type of Emodin-Induced Cell Death

**Figure 2:**
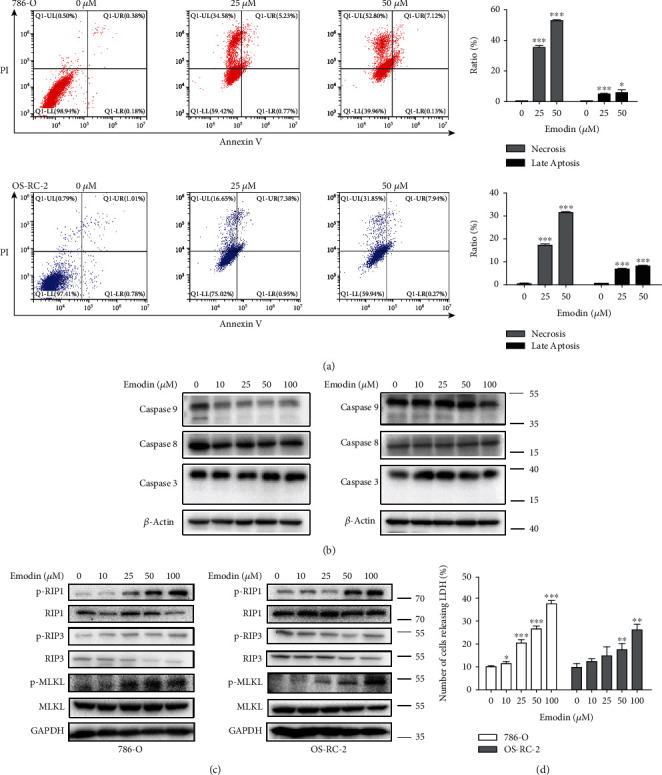
Emodin induced renal cancer cell death. (a) Different concentrations of emodin induce necrosis and apoptosis in 786-O and OS-RC-2 cells. Representative density plots of flow cytometric analysis on the fraction of dead cells 24 h after the indicated treatments detected with Annexin V/propidium iodide. The histogram represents the mean values of three independent experiments. (b) Caspase 9, caspase 8, and caspase 3 protein levels in the described cell lines were determined using western blot analysis. (c) p-RIP1, p-RIP3, and p-MLKL protein levels in the described cell lines were determined using western blot analysis. (d) LDH release tests in 786-O and OS-RC-2 cells after 24 h of treatment with emodin.

Flow cytometry assay with Annexin V-FITC and PI was used to detect the cell death. As determined by flow cytometry, emodin promoted the apoptosis and necrosis of OS-RC-2 and 786-Ocells in a dose-dependent manner (Figure 2(a)). The percentages of necrotic 786-O cells and OS-RC-2 cells treated with emodin for 24 h were 0.45%, 0.83% (0 *μ*M, control (CTL)), 35.38%, 17.23% (25 *μ*M), and 52.67%, 31.83% (50 *μ*M), respectively, while the percentages of late apoptotic 786-O cells and OS-RC-2 cells were 0.39%, 0.99% (CTL), 5.06%, 7.22%(25 *μ*M), and 5.84%, 8.26% (50 *μ*M), respectively. Moreover, significant changes of apoptosis-related protein expression, including caspase 9, caspase 8, and caspase 3, were not observed (Figure 2(b)). Interestingly, however, the phosphorylation levels of necroptosis-related proteins RIP1 and MLKL were significantly increased after emodin treatment (Figures 2(c) and 2(d)). Necroptosis is characterized by the deterioration of the cytoplasm membrane, which can be confirmed by LDH leakage [20, 21]. As determined by LDH cytotoxicity assay, the release of LDH markedly increased after emodin treatment (Figure 2(e)). Taken together, these data demonstrated that necroptosis, but not apoptosis, is the main cell death type induced by emodin.

### 3.3. ROS Was Involved in Necroptosis Induced by Emodin in Renal Cancer Cells

**Figure 3:**
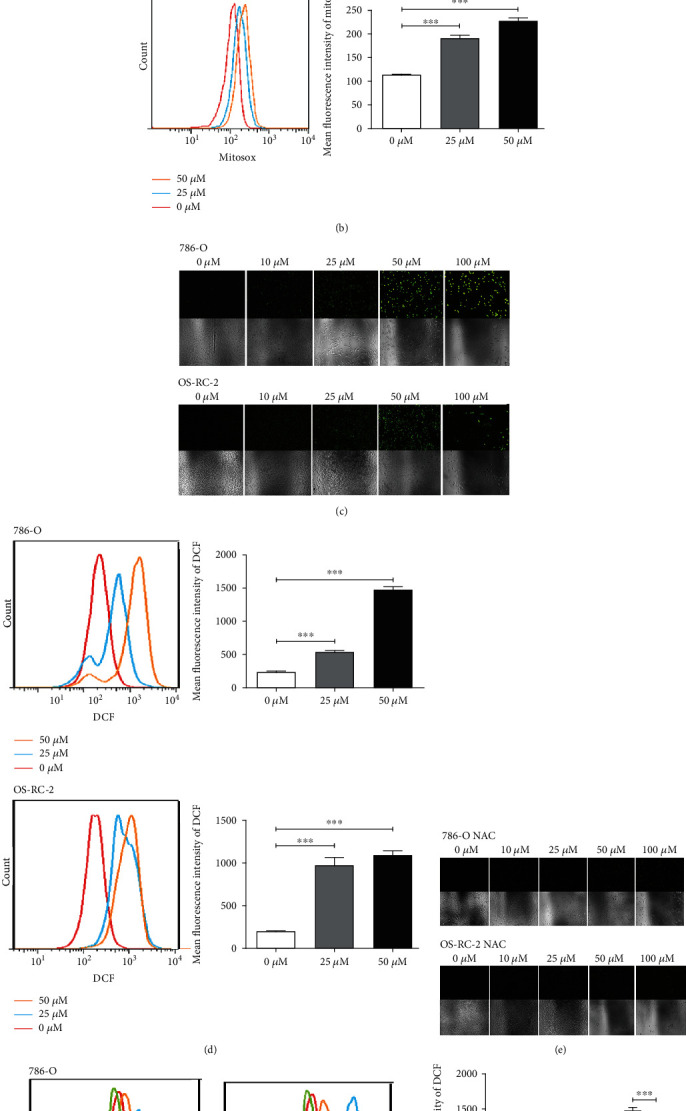
Emodin enhanced ROS production in renal cancer cells. (a) Fluorescence microscopy of emodin-treated 786-O and OS-RC-2 cells followed by 5 *μ*M Mitosox staining for 30 min (at 40x magnification). (b) Flow cytometry was used for the quantitative analysis of Mitosox. (c) Fluorescence microscopy of emodin-treated 786-O and OS-RC-2 cells followed by 10 *μ*M DCFHDA staining for 30 min (at 40x magnification). (d) Flow cytometry was used for the quantitative analysis of DCF. (e) 786-O and OS-RC-2 cells were pretreated with 10 mM NAC for 6 h, then fluorescence microscopy of emodin-treated 786-O and OS-RC-2 cells followed by 10 *μ*M DCFHDA staining for 30 min (at 40x magnification). (f) Flow cytometry was used for the quantitative analysis of DCF.

Previous studies have been reported that the molecular structure of emodin determined its property of transferring electrons and producing ROS [13]; moreover, necroptosis process is closely related to generation of ROS [22]. Therefore, to further investigate the mechanism of emodin-induced necroptosis in renal cancer cells, fluorescent dye MitoSox was utilized to specifically detect ROS levels in mitochondria. As shown in Figures 3(a) and 3(b), mitochondrial ROS levels increased in a dose-dependent manner following emodin treatment (Figures 3(a) and 3(b)). Meanwhile, consistent results were obtained by using fluorescent dye DCFH-DA to determine the intracellular overall ROS levels after emodin treatment (Figures 3(c) and 3(d)). These results indicated that emodin could increase ROS level in renal cancer cells.

**Figure 4:**
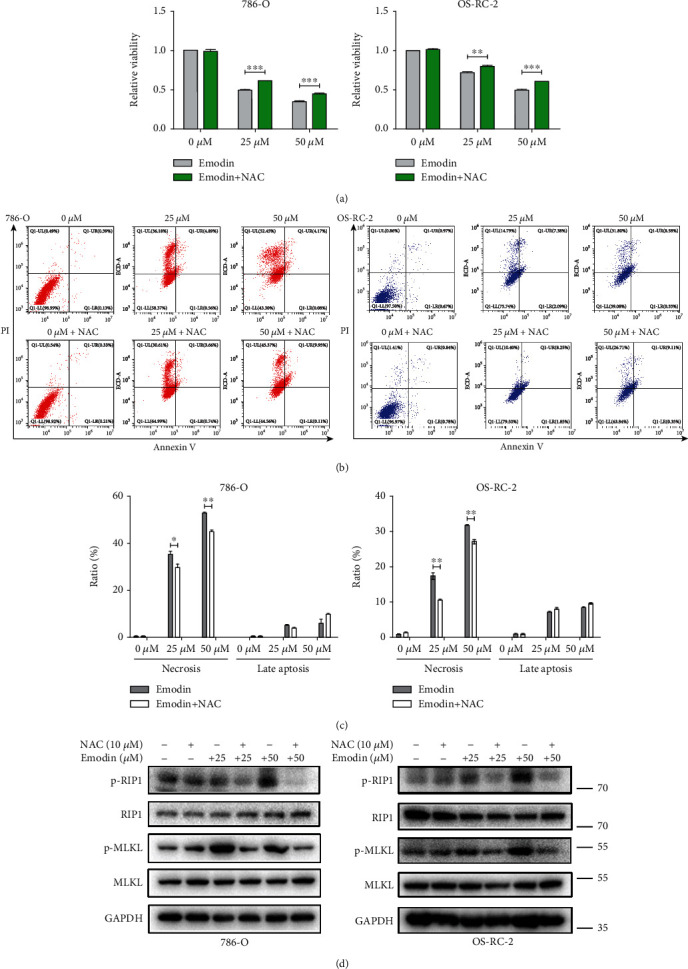
ROS was involved in necroptosis induced by emodin in renal cancer cells. (a) Emodin-induced cell viability inhibition of 786-O and OS-RC-2 cells could be reversed after pretreatment by 10 *μ*M NAC for 6 h. (b) NAC treatment could reduce emodin-induced necrosis. Representative density plots of flow cytometric analysis on the fraction of dead cells 24 h after the indicated treatments detected with Annexin V/propidium iodide. (c) The histogram represents the mean values of three independent experiments. (d) Western blot analysis of P-RIP1 and P-MLKL in 786-O and OS-RC-2 cells under the described condition.

To further address whether ROS production was a mediator of emodin-induced necroptosis, we examined the effects of antioxidant N-acetyl-cysteine (NAC) on necroptosis induced by emodin. As shown in Figures 3(e) and 3(f), NAC significantly suppressed the generation of ROS induced by emodin. Importantly, CCK-8 and flow cytometry assays indicated that emodin-induced cell viability inhibition and necroptosis, but not apoptosis, could be markedly reversed by NAC pretreatment in 786-O and OS-RC-2 cells (Figures 4(a)–4(c)). Meanwhile, the upregulated phosphorylation levels of RIP1 and MLKL induced by emodion were also reversed by NAC pretreatment (Figure 4(d)). Taken together, these results suggested that ROS was involved in necroptosis induced by emodin in renal cancer cells.

### 3.4. ROS Was Engaged in Glycolysis Inhibition Caused by Emodin in Renal Cancer Cells

**Figure 5:**
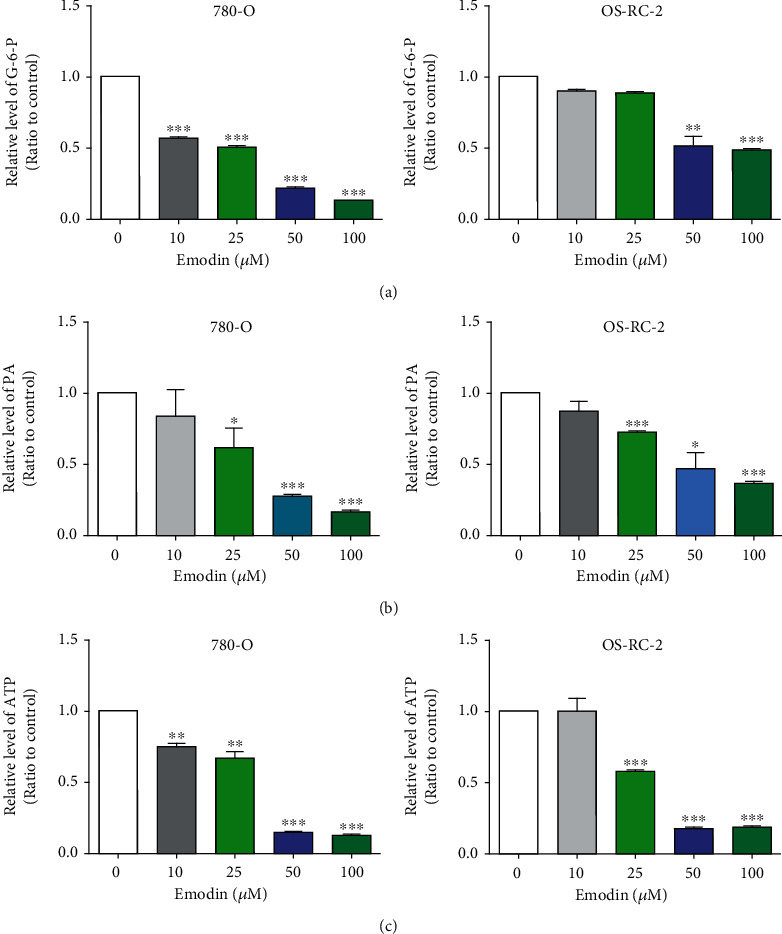
Emodin inhibited glycolysis in renal cancer cells in vitro. (a) Emodin induced significant reduction of glucose-6-phosphate in OS-RC-2 and 786-O renal cancer cells in a dose-dependent manner. (b) Pyruvate was decreased markedly by Emodin in OS-RC-2 and 786-O cells in dose-dependent manner. (c) The reduction of ATP induced by Emodin was dependent on the drug concentration.

Aerobic glycolysis, also known as the “Warburg effect,” is one of the most known hallmarks of cancer cells, meeting their metabolic requirements for supporting cell growth [23]. Previous study reported that oxidative stress resulting from the disrupted homeostasis between ROS production and scavenging can inhibit glycolysis [24]. Therefore, potent effects of emodin on ROS generation promoted us to investigate whether glycolysis process could be modulated by emodin. The levels of glucose-6-phosphate and pyruvate, the products of the first and the final irreversible steps of glycolysis, were detected after emodin treatment [25]. As shown in Figures 5(a) and 5(b), the levels of glucose-6-phosphate and pyruvate were both decreased in a dose-dependent manner after emodin treatment compared with the untreated cells. Moreover, the ATP produced primarily via glycolysis in cancer cells [26] was also obviously decreased in a dose-dependent manner after emodin treatment in renal cancer cells (Figure 5(c)). Together, these results suggested that emodin inhibited glycolysis in renal cancer cells in a concentration-dependent manner.

**Figure 6:**
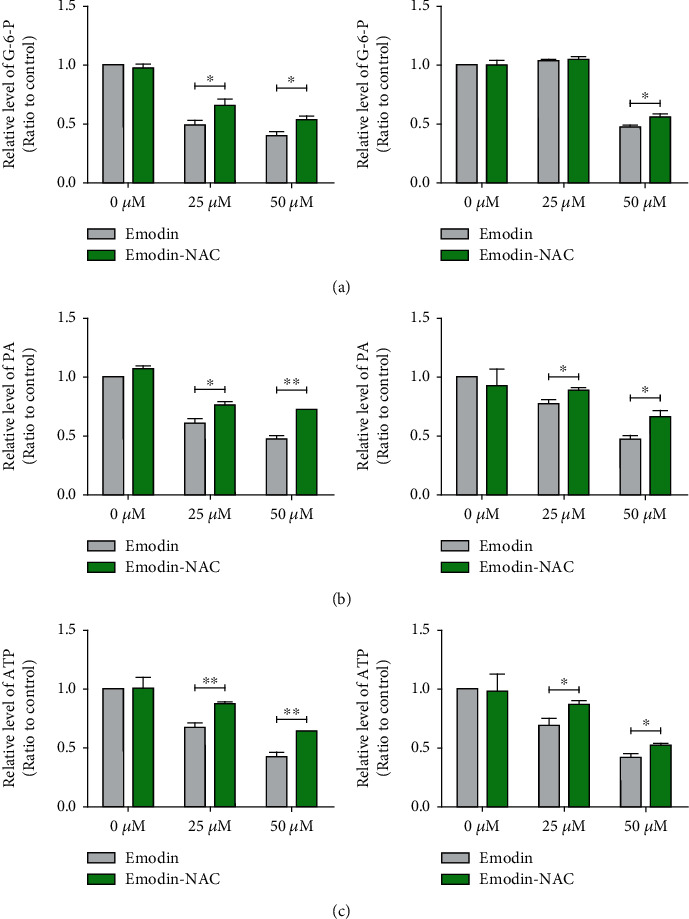
ROS was engaged in glycolysis inhibition caused by emodin in renal cancer cells. Pretreatment by 10 *μ*M NAC for 6 h could significantly rescued emodin-induced decreases of glucose-6-phosphate (a), pyruvate (b), and ATP (c).

To further confirm whether the inhibition of glycolysis induced by emodin depends on the increase of ROS, the antioxidant N-acetyl-cysteine (NAC) was used to reduce the level of ROS and retested the progress of glycolysis. The results showed that decreases of glucose-6-phosphate, pyruvate, and ATP induced by emodin were significantly reversed by NAC (Figure 6). Thus, these data demonstrated that ROS was engaged in emodin-induced glycolysis suppression.

### 3.5. Emodin Induced Necroptosis through Increased ROS in a JNK-Dependent Pathway

**Figure 7:**
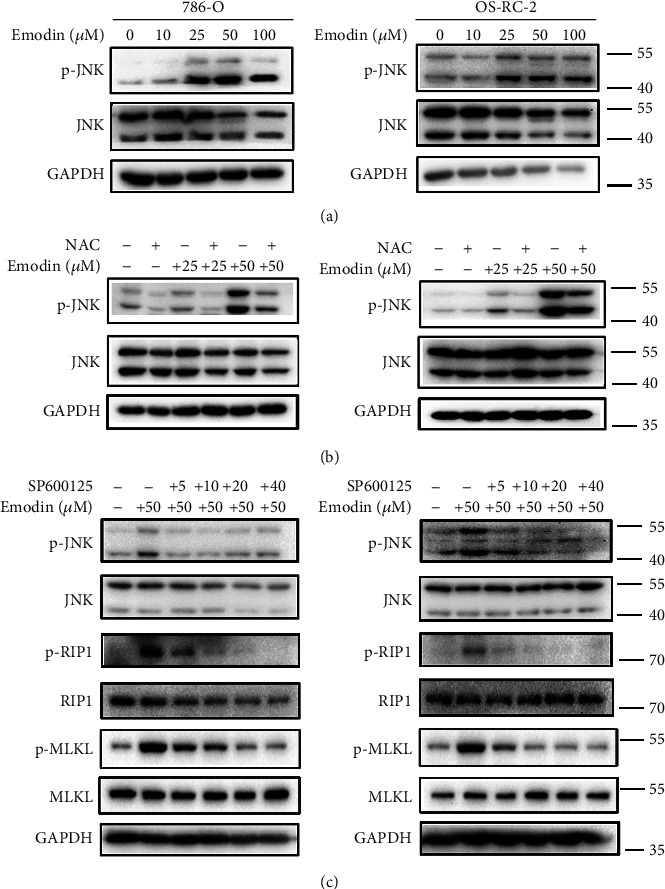
Emodin induced necroptosis through increased ROS in a JNK-dependent pathway. (a) The level of p-JNK was examined by western blot in 786-O and OS-RC-2 cells treatment with different concentrations of emodin (0, 10, 25, 50, and 100 *μ*M, respectively) for 24 h. (b) Western blot analysis of p-JNK level in 786-O and OS-RC-2 cells. The cells were treated with 0, 25, and 50 *μ*M emodin for 24 h, respectively, with or without NAC pretreatment for 6 h. (c) Effect of JNK inhibitor SP600125 on p-JNK, p-RIP1, and p-MLKL by western blot analysis. 786-O and OS-RC-2 cells were treated with 50 *μ*M emodin for 24 h with SP600125 pretreatment (0, 5, 10, 20, and 40 *μ*M, respectively) for 3 h.

Previous studies have demonstrated that stress on cells arises from the generation of free radicals can activate JNK [27]. We found that as shown in Figure 7(a), emodin significantly activated JNK signaling as indicated by the increasing level of JNK phosphorylation. Importantly, activation of JNK signaling induced by emodin could be markedly rescued upon the ROS scavenging (Figure 7(b)). JNK pathway plays important roles in modulating apoptosis [28, 29], but its biological importance for necroptosis is not clear. To determine whether emodin induced necroptosis dependent on the activation of JNK pathway, the JNK inhibitor SP600125 was utilized. As shown in Figure 7(c), the emodin-induced increasing phosphorylation of JNK could be reversed by the SP600125; more importantly, that of RIP1 and MLKL were also significantly attenuated. Taken together, these data suggested that emodin induces necroptosis through ROS-mediated activation of JNK signaling pathway.

### 3.6. Emodin Inhibited Glycolysis by Downregulation GLUT1 through ROS-Mediated PI3K/AKT Signaling Pathway

**Figure 8:**
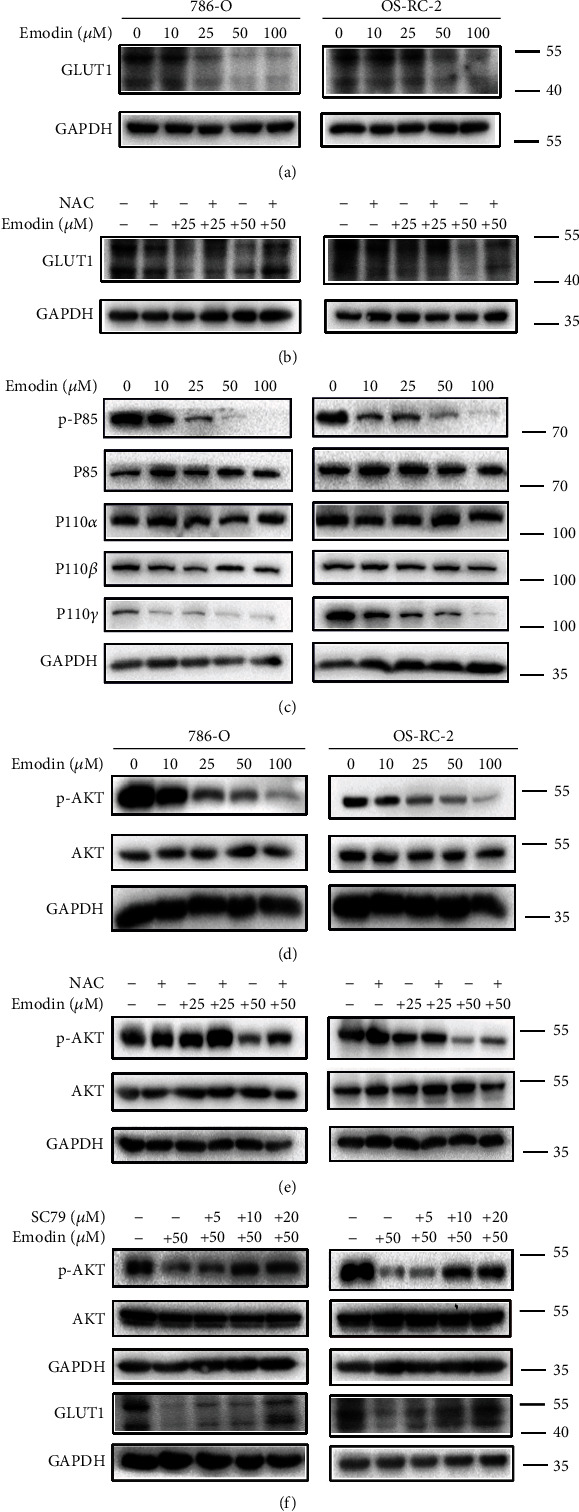
Emodin inhibited glycolysis by downregulation of GLUT1 through ROS-mediated PI3K/AKT signaling pathway. (a, c, d) 786-O and OS-RC-2 cells were treated with different concentrations of emodin (0, 10, 25, 50, and 100 *μ*M, respectively) for 24 h. The expression levels of GLUT1, p-P85, P85, P110*α*, P110*β*, P110*γ*, p-AKT, and AKT were tested by western blot. (b, e) Western blot analysis of GLUT1 and p-AKT levels in 786-O and OS-RC-2 cells. The cells were treated with 0, 25, and 50 *μ*M emodin for 24 h, respectively, with or without NAC pretreatment for 6 h. (f) Effect of AKT activator SC79 on p-AKT and GLUT1 by western blot analysis. 786-O and OS-RC-2 cells were treated with 50 *μ*M emodin for 24 h with SC79 pretreatment (0, 5, 10, 20, and 40 *μ*M, respectively) for 3 h.

Glucose translocation across the plasma membrane, the critical initial step during glycolysis process, was triggered by carriers belonging to the facilitative glucose transporter (GLUT) family. GLUT1 is the member with the highest affinity for glucose, frequently upregulated in cancers, is responsible for the basal uptake of glucose in all tissues [30], likely contributing to the avid uptake of glucose even when its availability is becoming insufficient because of the continuous growth of the tumor [31]. Therefore, membrane proteins was extracted and the expression of GLUT1 was examined after emodin treatment. As shown in Figures 8(a) and 8(b), the expression of GLUT1 significantly decreased after emodin treatment, and this phenomenon could be partly reversed by ROS scavenging. It has been reported that AKT activation is relative to maintaining glucose homeostasis [32]; then we started to focus on PI3K/AKT signaling and found that the decreased phosphorylation levels of p85, p110 *γ*, and AKT were also dependent on emodin-induced ROS generation (Figures 8(c)–8(e)). To investigate the relationship between PI3K/AKT pathway and GLUT1 expression, AKT agonist SC79 was utilized. As shown in Figure 8(f), the emodin-induced GLUT1 downregulation was rescued by the SC79-mediated AKT activation. Taken together, these results demonstrated that emodin inhibits glycolysis by downregulating GLUT1 dependent on ROS-mediated inactivation of PI3K/AKT signaling pathway. These data were also consistent with the results in Figure 6. Therefore, these results suggested that emodin-induced downregulation of GLUT1 is mediated by ROS generation.

## 4. Discussion

Emodin, a naturally occurring anthraquinone, present in the roots and barks of numerous plants, is an active ingredient of various natural plant including *Rheum officinale* and *Polygonum cuspidatum* medicine. It plays important roles in anti-inflammatory, antibacterial, diuretic, immunosuppressive, and antitumor activities [33–35]. However, its role in RCC remains obscure. In our study, we preliminarily confirmed that emodin can effectively kill renal cancer cells, but with very lower toxicity to noncancerous renal tubular epithelial cell. At the same time, we detected that the levels of ROS increased following emodin treatment in a dose-dependent manner.

A large number of reports showed that ROS plays an important regulatory role in the growth of tumor cells [36]. Excessive ROS is detrimental to the survival of cancer cells, and lots of antitumor drugs induce apoptosis and autophagy by increasing the levels of ROS in cancer cells [37, 38]. Interestingly, the molecular structure of emodin is similar to that of DMNQ, an agent that generates ROS intracellularly because its property of quinone and derived semiquinone, like mitochondrial ubiquinone, allows it to transfer electrons to produce ROS [13]. Therefore, we hypothesized that the antirenal cancer effect of emodin is closely related to the production of ROS.

It has been well established that excessive ROS triggers downstream cellular and molecular events such as alterations of mitochondrial function and signal transduction leading to apoptotic cell death [34]. But in our experimental results, we found that it is necrosis rather than apoptosis, which is the main cell death type induced by emodin. Necrosis has been considered a random, passive, genetically unregulated process for a long time in the past years; however, accumulating evidences have confirmed that necrosis is also regulated by some necrotic-associated genes but is not dependent on the caspase signaling pathway. Necroptosis is one of the most well-studied forms of necrosis. Activation of the necroptosis involves the formation of a complex named necrosome containing RIP3 and its family number, RIP1, as well as the recruitment and phosphorylation of mixed lineage kinase domain-like protein (MLKL), which triggers its oligomerization and plasma membrane localization, eventually leading to the rupture of the cell membrane and cell death [39–41]. Therefore, RIP1, RIP3, and MLKL are the core molecules in the necroptosis signaling pathway. We examined the expression of these three proteins and found that the phosphorylation levels of RIP1 and MLKL were significantly increased after emodin treatment.

Zhang et al. verified that RIP1 is sense to ROS via modification of three crucial cysteine residues, and its autophosphorylation on S161 is induced subsequently. This phosphorylation event allows efficient recruitment of RIP3 to RIP1 to form a functional necrosome [42]. To verify whether activation of necroptosis is associated with emodin-induced increases in ROS, we treated renal cells with ROS scavenger NAC. And we found that NAC significantly rescued emodin-induced necroptosis and downregulated the expression of the p-RIP1. These results show that ROS was involved in necroptosis induced by emodin in renal cancer cells.

There is still a question how ROS is associated with necroptosis. As we know, Jun-N-terminal kinases were activated to various stress conditions in mammals [43]. Previous studies also demonstrated that stress on cells arises from the generation of free radicals can activate JNK [27]. Then we tested the phosphorylation level of JNK and found that the level of p-JNK was obviously elevated following emodin treatment in a dose-dependent manner. NAC also inhibited the activation of JNK, which suggested that ROS is necessary to JNK activation. Furthermore, the JNK inhibitor SP600125 suppressed the phosphorylation of RIP1 and MLKL. These results demonstrate that emodin could induce necroptosis through increased ROS in a JNK-dependent pathway.

The Warburg effect, also known as aerobic glycolysis, is the process by which cancer cells convert glucose into lactic acid under aerobic conditions to quickly gain energy [23]. Aerobic glycolysis is the main way for cancer cells to gain energy, so the blocking of the glycolytic pathway can effectively inhibit the growth of cancer cells. It has been reported that oxidative stress resulting from the disrupted homeostasis between ROS production and scavenging can inhibit glycolysis [24]. In previous experiments, we have confirmed that emodin can increase ROS production, so we next explore the effect of emodin on glycolysis. Our experiment detected that emodin induced the downregulation in the levels of glucose-6-phosphate and pyruvate which, respectively, represented the product of the first and the final irreversible steps of glycolysis. Moreover, the ATP produced primarily via glycolysis in cancer cells was also obviously decreased by emodin in these renal cancer cells. These results suggested that emodin inhibited glycolysis in renal cancer cells. Interestingly, clearing ROS can also reverse the inhibition of glycolysis, which proved that ROS plays an important role in this suppression process.

But what kind of mechanism does ROS affect the glycolysis process of kidney cancer cells? To answer this question, we conducted further research. Glucose translocation across the plasma membrane, the critical initial step in glycolysis, occurs through carriers belonging to the facilitative glucose transporter (GLUT) and the sodium-coupled glucose cotransporter (SGLT) proteins families. Among them, GLUT1 is the member with the highest affinity for glucose and is responsible for the basal uptake of glucose in all tissues. GLUT1 is frequently found upregulated in cancers [30], likely contributing to the avid uptake of glucose even when its availability is becoming insufficient because of the continuous growth of the tumor [31]. During our research, we found that the expression of GLUT1 decreased significantly after emodin treatment, and this decrease could be improved to some extent after ROS removal. This was consistent with the glycolytic appearance we measured before. Therefore, we believed that emodin-induced downregulation of GLUT1 is related to ROS.

Insulin, downstream of Akt activation, promotes glucose uptake into fat and muscle cells to lower postprandial blood glucose, an enforced change in cellular metabolism to maintain glucose homeostasis [32]. Therefore, we speculated whether the decreased expression of GLUT1 was related to the AKT pathway. Then we tested the four subunits of PI3K and the phosphorylation level of AKT and found that the phosphorylation level of P85 and AKT decreased with the increase of emodin concentration. However, ROS scavenger NAC could restore the phosphorylation level of AKT to a certain extent. Next, we used AKT activator SC79 to activate the level of phosphorylation inhibited by emodin. We found that as the level of AKT phosphorylation increased, the expression of GLUT1 also similarly recovered. Thus, we suggested that emodin inhibited glycolysis by downregulating GLUT1 through ROS-mediated PI3K/AKT signaling pathway.

## 5. Conclusions

**Figure 9:**
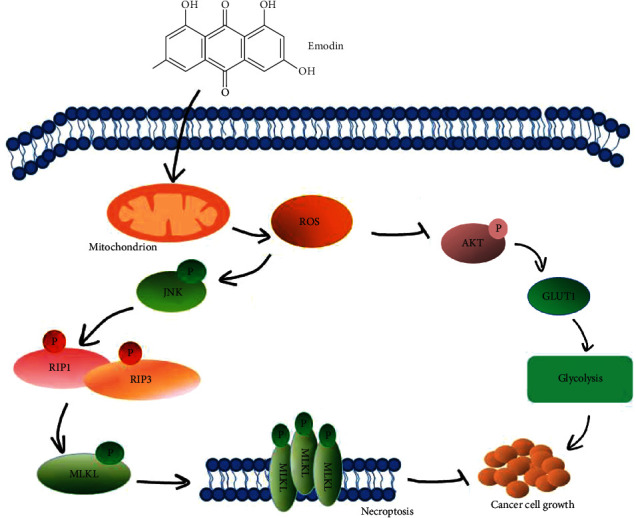
Schematic representation of necroptotic signaling stimulated by emodin in human renal cancer cells. Emodin produces a large amount of ROS by affecting the mitochondrial electron transport chain. On the one hand, ROS can inhibit aerobic glycolysis to suppress tumor cell proliferation, and on the other hand, ROS induces cell necroptosis by activating JNK signaling to phosphorylate the death-executing protein MLKL.

The PI3K/AKT pathway plays a key role in many cancers [44], especially in renal cancer, and the changes in PI3K/AKT pathway account for 28% [45]. JNK signaling is involved in chemotherapeutic resistance and immune evasion; however, renal cancer is always resistant to chemotherapy and weakly sensitive to immunotherapeutic agents [46]. Therefore, PI3K/AKT and JNK are two vital pathways in renal cancer. In this study, we found that emodin effectively induces necroptosis in renal cancer cells without significant cytotoxicity to noncancerous renal tubular epithelial cell, and emodin induces necroptosis through ROS-mediated activation of JNK signaling pathway. Moreover, further studies demonstrated that emodin also inhibits glycolysis by downregulation of GLUT1 dependent on ROS-mediated inactivation of the PI3K/AKT signaling pathway (Figure 9). These potential mechanisms implicated that emodin or other ROS-producing agents may bring benefits for patients with JNK- or PI3K/AKT-dysregulated renal cancer.

## Data Availability

All the data used to support the findings of this study are included within the article and the supplementary materials.

## Supplementary Materials

Supplementary Materials Figure S1: NAC could improve the survival of renal cancer cells after emodin treatment. Figure S2: emodin had no significant effects on HK-2 cells. Figure S3: histogram of the key genes expression of apoptosis and necroptosis after emodin treatment. Figure S4: histogram of phosphorylation levels of RIP1 and MLKL after pretreatment with 10*μ*MNAC for 6 hours and then treatment with emodin for 24 hours. Figure S5: ROS was involved in emodin induced necroptosis in a JNK-dependent pathway (the histogram of Figure 7). Figure S6: emodin inhibited glycolysis by downregulation of GLUT1 through ROS-mediated PI3K/AKT signaling pathway (the histogram of Figure 8).
