# Supplementary material for: Emodin Induced Necroptosis and Inhibited Glycolysis in the Renal Cancer Cells by Enhancing ROS
Source: Oxid Med Cell Longev. 2021 Jan 19;2021:8840590. doi: 10.1155/2021/8840590 (PMC7837784; doi:10.1155/2021/8840590)
Supplement: Supplementary Materials — Figure S1: NAC could improve the survival of renal cancer cells after emodin treatment. Figure S2: emodin had no significant effects on HK-2 cells. Figure S3: histogram of the key genes expression of apoptosis and necroptosis after emodin treatment. Figure S4: histogram of phosphorylation levels of RIP1 and MLKL after pretreatment with 10μMNAC for 6 hours and then treatment with emodin for 24 hours. Figure S5: ROS was involved in emodin induced necroptosis in a JNK-dependent pathway (the histogram of Figure 7). Figure S6: emodin inhibited glycolysis by downregulation of GLUT1 through ROS-mediated PI3K/AKT signaling pathway (the histogram of Figure 8). [file 8840590.f1.docx]

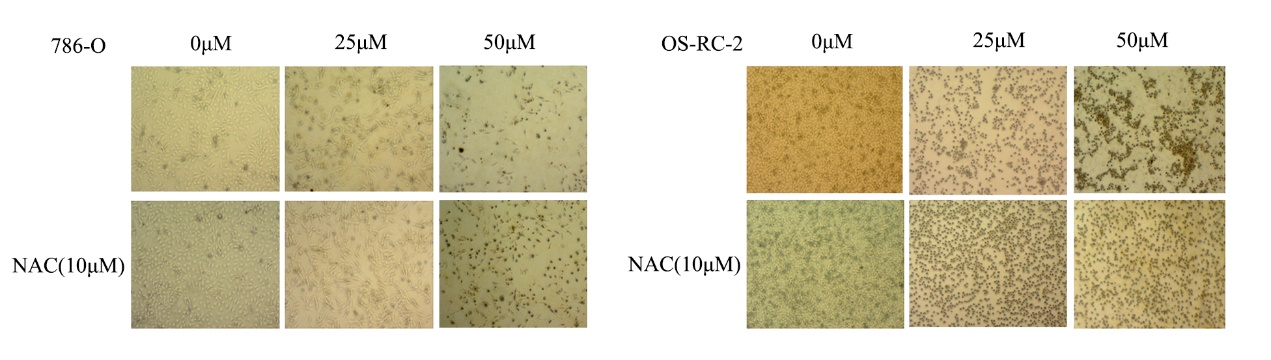


Fig.S1 NAC could improve the survival of renal cancer cells after emodin treatment.

Morphology of 786-O and OS-RC-2 cells treated with different concentrations of emodin (0, 25 and 50μ M, respectively ) for 24 h with or without NAC pretreatment for 6 h.


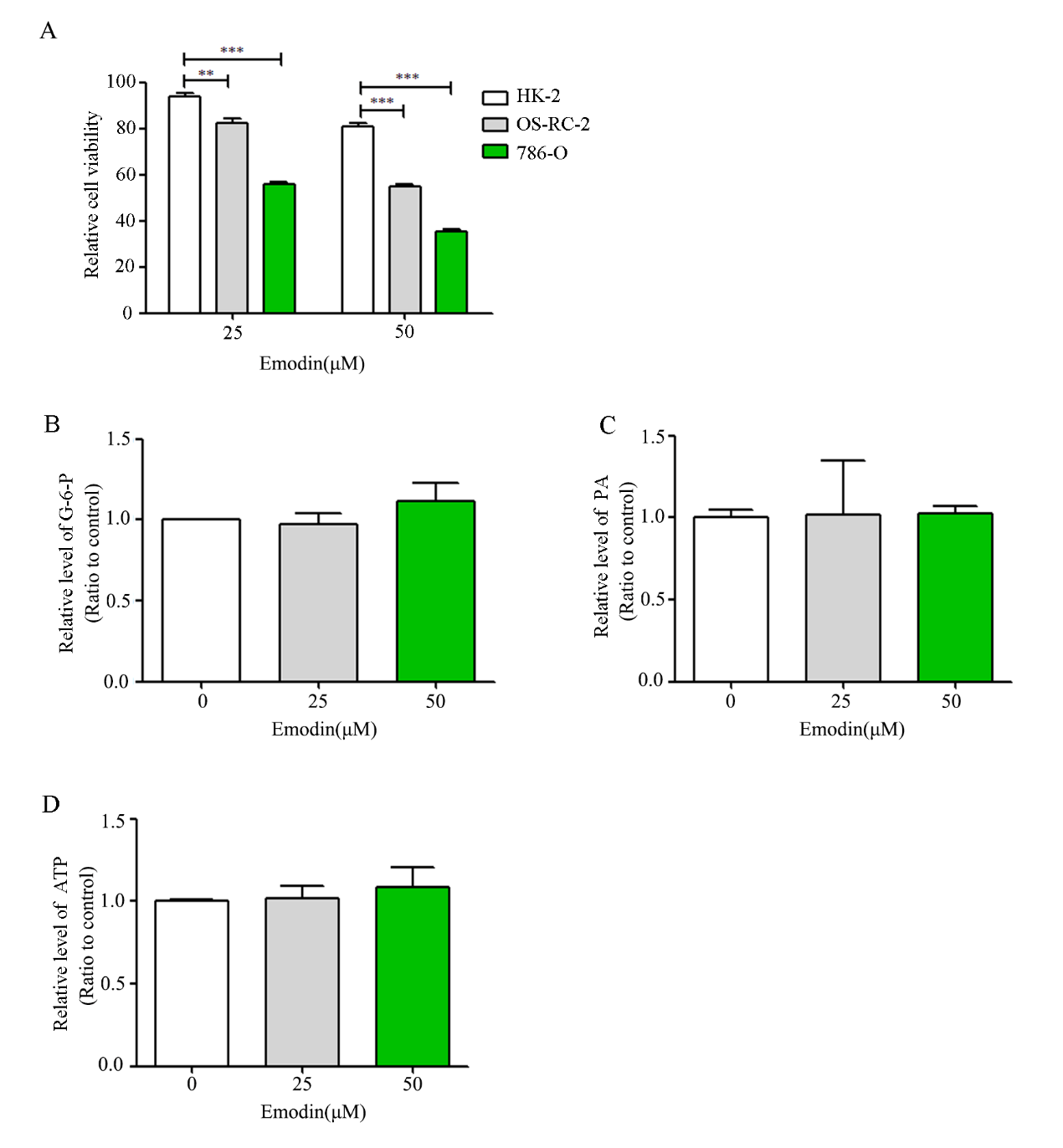


Fig.S2 Emodin had no significant effects on HK-2 cells.

A. Changes of HK-2,786-O and OS-RC-2 cells viability after 25µM, 50µM emodin treatment.

B. Relative level of G-6-P after emodin treatment.

C. Relative level of PA after emodin treatment.

D. Relative level of ATP after emodin treatment.


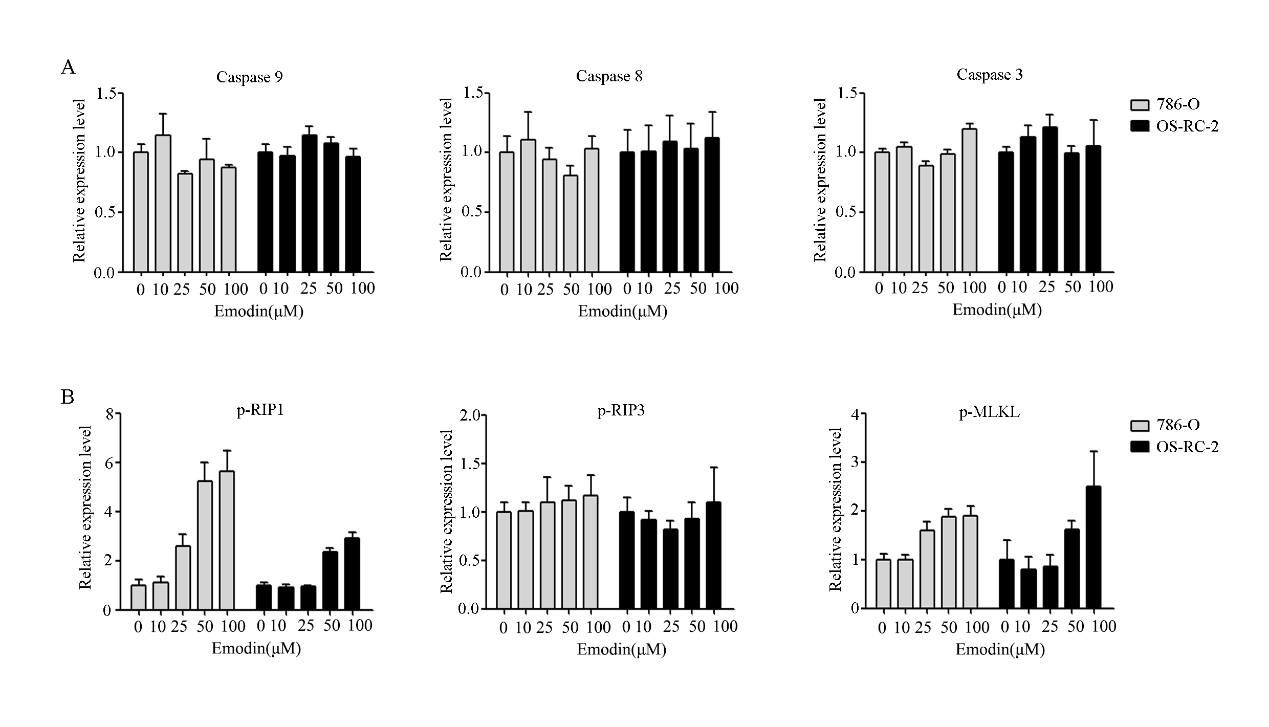


Fig.S3 Histogram of the key genes expression of apoptosis and necroptosis after emodin treatment.

1. The protein expression of Caspase9, Caspase8 and Caspase3 were normalized with β-actin and compared with the control group(0µM).
2. The protein expression of p-RIP1, p-RIP3 and p-MLKL were normalized with their respective total protein and compared with the control group(0µM).


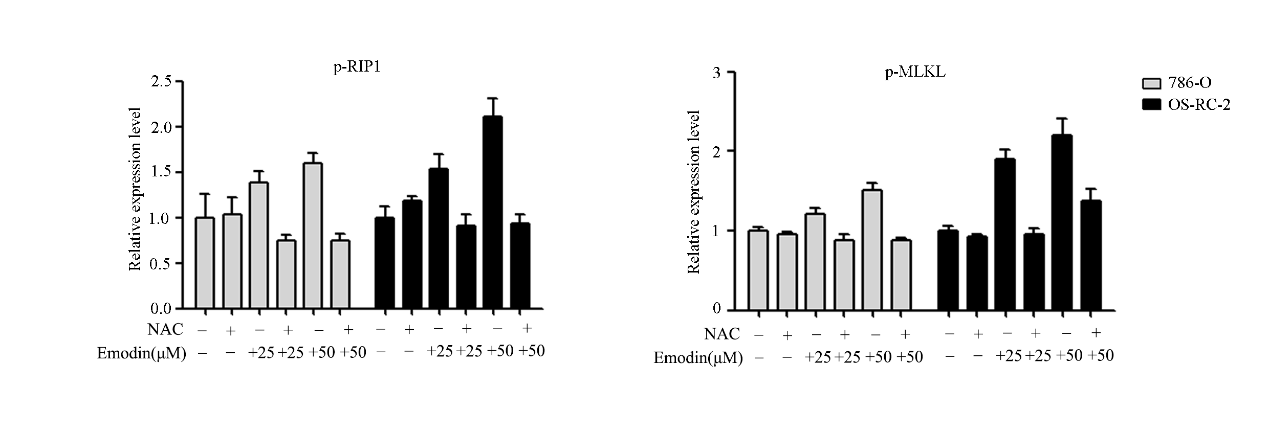


Fig. S4 Histogram of phosphorylation levels of RIP1 and MLKL after pretreatment with 10μMNAC for 6 hours and then treatment with emodin for 24 hours.

The protein expression of p-RIP1 and p-MLKL were normalized with their respective total protein and compared with the control group (0µM emodin and 0µM NAC).


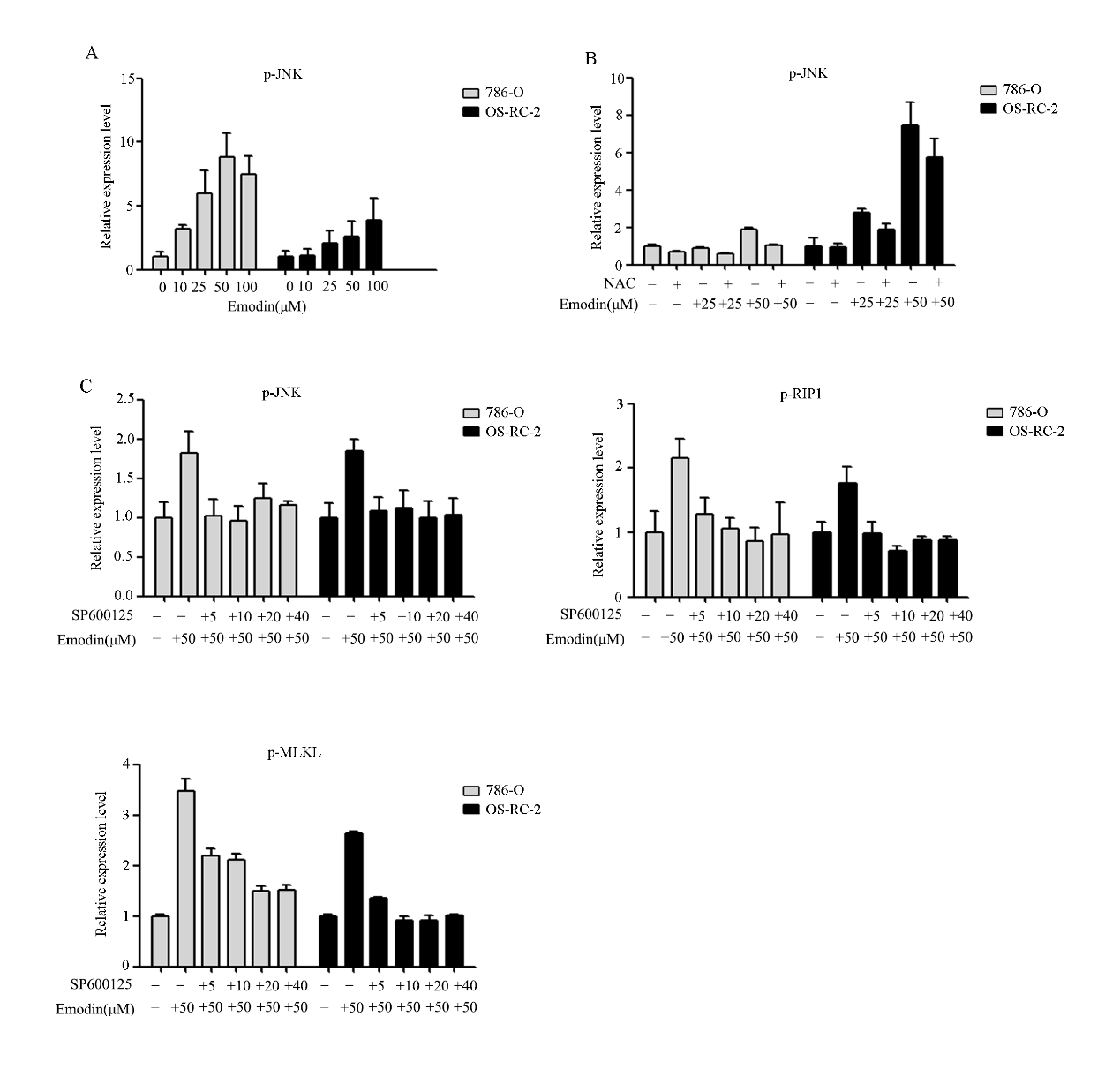


Fig. S5 ROS was involved in emodin induced necroptosis in a JNK dependent pathway (the histogram of Figure 7).

1. The protein expression of p-JNK was normalized with GAPDH and compared with the control group(0µM) after treatment with emodion.
2. The protein expression of p-JNK was normalized with JNK total protein and compared with the control group(0µM emodin and 0µM NAC) after pretreatment with 10μMNAC for 6 hours and then treatment with emodin for 24 hours.
3. The protein expression of p-JNK ,p-RIP1 and p-MLKL were normalized with their respective total protein and compared with the control group(0µM emodin and 0µM SP600125) after pretreatment with JNK inhibitor for 3 hours and then treatment with emodin for 24 hours.


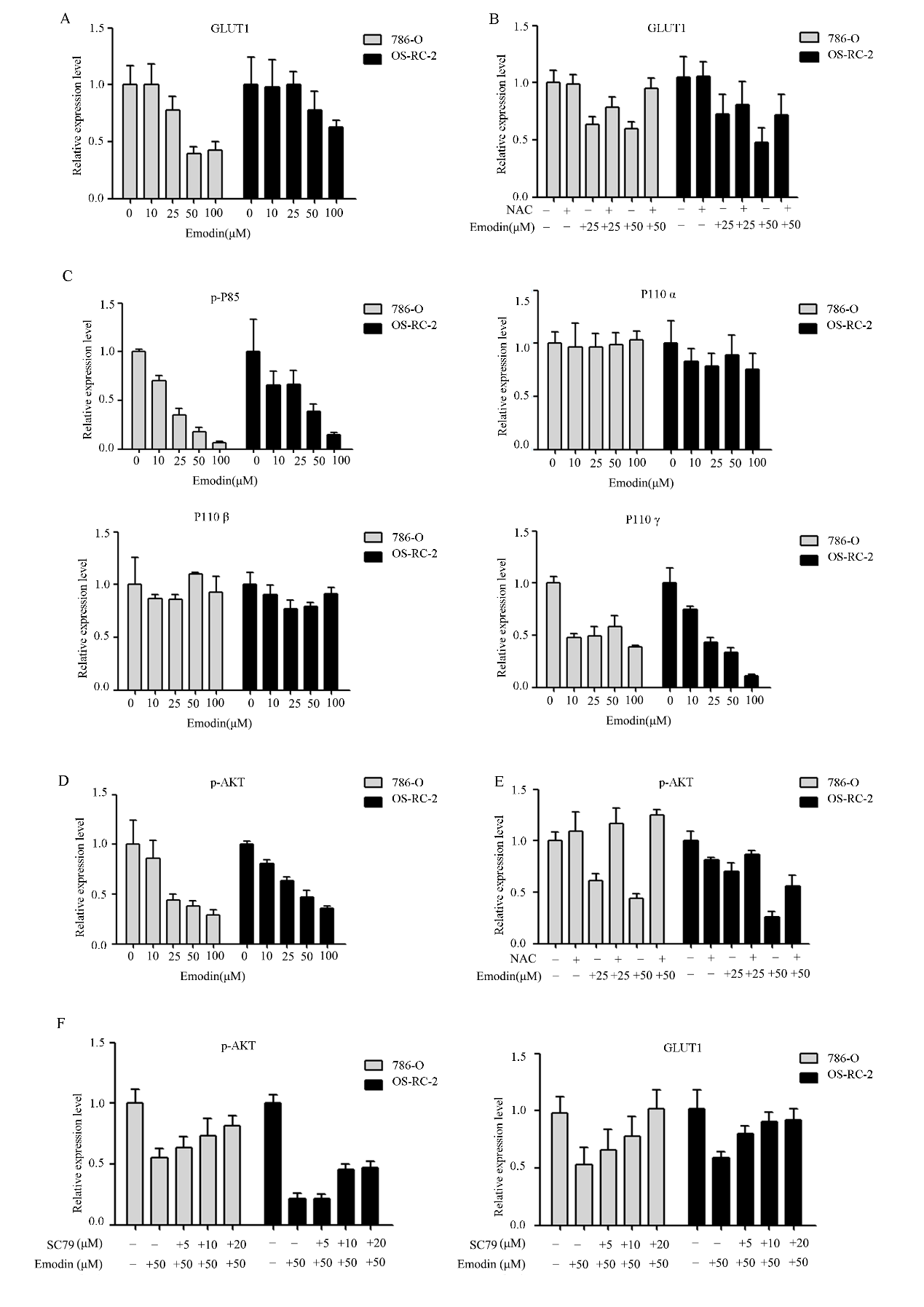


Fig.S6 Emodin inhibited glycolysis by down-regulation GLUT1 through ROS-mediated PI3K/AKT signaling pathway (the histogram of Figure 7).

1. The protein expression of GLUT1 was normalized with GAPDH and compared with the control group(0µM) after treatment with emodion.
2. The protein expression of GLUT1 was normalized with GAPDH and compared with the control group(0µM emodin and 0µM NAC) after pretreatment with 10μMNAC for 6 hours and then treatment with emodin for 24 hours.
3. The protein expression of p-P85 was normalized with p85 total protein, while the protein expression of P110α, P110β and P110γ were normalized with GAPDH. And all the proteins were compared with the control group(0µM) after treatment with emodion.
4. The protein expression of p-AKT was normalized with AKT total protein and compared with the control group(0µM) after treatment with emodion.
5. The protein expression of p-AKT was normalized with AKT total protein and compared with the control group(0µM emodin and 0µM NAC) after pretreatment with 10μMNAC for 6 hours and then treatment with emodin for 24 hours.
6. The protein expression of p-AKT was normalized with AKT total protein, while the protein expression of GLUT1 was normalized with GAPDH. And all the proteins were compared with the control group(0µM emodin and 0µM SC79) after pretreatment with AKT activator for 3 hours and then treatment with emodin for 24 hours.
